# Design and characterization of structured protein linkers with differing flexibilities

**DOI:** 10.1093/protein/gzu043

**Published:** 2014-10

**Authors:** Joshua S. Klein, Siduo Jiang, Rachel P. Galimidi, Jennifer R. Keeffe, Pamela J. Bjorkman

**Affiliations:** 1 Division of Biology and Biological Engineering, California Institute of Technology, Pasadena, CA 91125, USA; 2 Howard Hughes Medical Institute, California Institute of Technology, Pasadena, CA 91125, USA

**Keywords:** fusion proteins, hydrodynamic radius, linker design, size-exclusion chromatography

## Abstract

Engineered fusion proteins containing two or more functional polypeptides joined by a peptide or protein linker are important for many fields of biological research. The separation distance between functional units can impact epitope access and the ability to bind with avidity; thus the availability of a variety of linkers with different lengths and degrees of rigidity would be valuable for protein design efforts. Here, we report a series of designed structured protein linkers incorporating naturally occurring protein domains and compare their properties to commonly used Gly_4_Ser repeat linkers. When incorporated into the hinge region of an immunoglobulin G (IgG) molecule, flexible Gly_4_Ser repeats did not result in detectable extensions of the IgG antigen-binding domains, in contrast to linkers including more rigid domains such as β2-microglobulin, Zn-α2-glycoprotein and tetratricopeptide repeats. This study adds an additional set of linkers with varying lengths and rigidities to the available linker repertoire, which may be useful for the construction of antibodies with enhanced binding properties or other fusion proteins.

## Introduction

Fusion proteins are engineered biomolecules containing parts from two or more genes synthesized as a single multi-functional construct. These have been critical in many areas of biological research including affinity purification (Lichty *et al*., 2005) and protein stabilization for structure determination (Zou *et al*., 2012). Bi-specific fusion proteins have also been utilized as biopharmaceuticals, with an active drug domain fused to a carrier domain, allowing for the drug's proper transport (Chen *et al*., 2013). Such proteins have been designed to penetrate epithelial membranes including the blood–brain barrier, as well as to target a specific cell population (Pardridge, 2010). Due to the modularity of protein domains in the generation of functional constructs, fusion proteins will likely have increasing importance in research and drug design.

The successful construction of fusion proteins relies on the proper choice of a protein linker as direct fusion of two domains can lead to compromised biological activity (Bai *et al*., 2005; Zhang *et al*., 2009). Several studies have utilized existing databases to compile and characterize linkers in naturally occurring multi-domain proteins (Argos, 1990; George and Heringa, 2002). These studies have yielded amino acid sequence propensities for natural linkers of various sizes and lengths, as well as information on rigidity and secondary structure. This information has helped the empirical design of linkers that are customized for particular applications.

Linkers can be classified into three groups: flexible, rigid and cleavable (Chen *et al*., 2013). Flexible linkers are generally composed of small, non-polar or polar residues such as Gly, Ser and Thr. The most common is the (Gly_4_Ser)*_n_* linker (Gly–Gly–Gly–Gly–Ser)*_n_*, where *n* indicates the number of repeats of the motif. Polyglycine linkers have also been evaluated, but the addition of a polar residue such as serine can reduce linker–protein interactions and preserve protein function. Due to their flexibility, these linkers are unstructured and thus provided limited domain separation in a previous study (Evers *et al*., 2006). As a result, more rigid linkers including polyproline motifs (Schuler *et al*., 2005) and an all α-helical linker A(EAAAK)*_n_*A (Arai *et al*., 2001) have been developed.

We are interested in using relatively rigid protein linkers to separate anti-HIV binding proteins at distances that would permit bi- or multi-valent binding to HIV Env glycoproteins with the objective of creating reagents capable of cross-linking epitopes within a single Env trimer (intra-spike cross-linking). Such reagents would take advantage of avidity effects to minimize HIV's ability to evade neutralizing antibodies by rapidly mutating to lower the affinity between the HIV epitopes and the antigen recognition fragment (Fab) of the antibody (Klein *et al*., 2009). Although the architecture of the HIV spike trimer does not permit intra-spike cross-linking by most natural antibodies (Zhu *et al*., 2006; Klein and Bjorkman, 2010), it may be possible to create reagents capable of bivalent binding to an HIV Env trimer by fusing two identical reagents or two different reagents with an appropriate length linker. Here we report the design, construction and characterization of a series of structured protein linkers incorporating both rigid and flexible domains that can be used to achieve a variety of different desired separations. The linkers were incorporated into the hinge region of an intact immunoglobulin G (IgG) antibody and evaluated for their relative lengths and rigidities by dynamic light scattering (DLS).

## Methods

### Plasmid construction and protein purification

Genes encoding designed linkers were synthesized (Blue Heron Bio) with restriction sites for the enzymes NheI (5′-end) and either NgoMIV or HindIII (3′-end). These sites were also introduced into the gene encoding the heavy chain of the HIV-neutralizing antibody b12 (Roben *et al*., 1994) such that the insert would be located between hinge region residues His235 and Thr236. Constructs encoding the b12 heavy chain gene with a linker inserted in the hinge region were subcloned into the pTT5 mammalian expression vector. The b12-linker IgGs were expressed transiently in HEK-6E cells by co-transfecting the b12-linker heavy chain genes with the b12 light chain gene as described (Diskin *et al*., 2011).

IgG-linker fusion constructs were purified by protein A affinity chromatography (GE Healthcare) followed by purification and analysis by size-exclusion chromatography (SEC) using a Superdex 200 10/300 GL column (GE Healthcare) in phosphate-buffered saline, 0.05% w/v sodium azide, pH 7.4.

### Dynamic light scattering

Fractions corresponding to the center of the SEC elution peak were concentrated using Amicon Ultra-15 Centrifugal Filter Units (Millipore) with a molecular weight cutoff of 100 kDa to a volume of 80–400 μl and concentrations of 0.5–1 mg/ml. Concentration differences within this range were not observed to affect the hydrodynamic radius values determined by DLS (data not shown). Sample sizes ranging from 80 to 350 µl were loaded into a disposable cuvette, and measurements were performed on a DynaPro® NanoStar™ (Wyatt Technology) using manufacturer's suggested settings. A fit of the second-order autocorrelation function to a globular protein model was used to derive the hydrodynamic radius.

## Results and discussion

### Design and identity of designed linkers

**Fig. 1.**
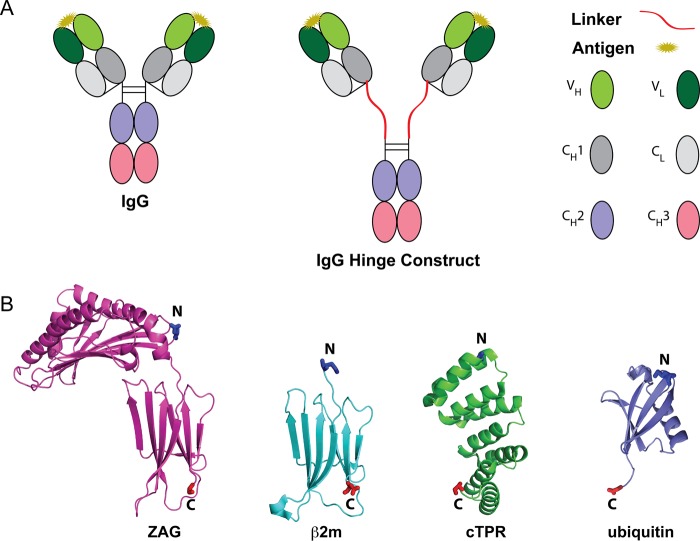
(A) Schematic of wild-type IgG (left) and IgG with a designed linker in its hinge region (middle). IgG domains are color coded as shown in the right panel. (B) Ribbon diagrams for domains used in structured linkers shown to scale (pdb codes: ZAG (1ZAG), β2m (1LDS), cTPR (2FO7), ubiquitin (1UBQ)). The cTPR structure shown contains eight tandem repeats. N- and C-terminal residues are shown as sticks, color-coded blue for the N-terminus and red for the C-terminus.

In order to design potential structured linkers, we surveyed the Protein Data Bank (PDB) to find structures that were relatively elongated and rigid, or represented small globular proteins. We chose Zn-α2-glycoprotein (ZAG; PDB code: 1ZAG) as an example of a relatively elongated and rigid structure (Sanchez *et al*., 1999), and β2-microgloblin (β2m; PDB code: 1LDS) and ubiquitin (Ub; PDB code: 1UBQ) as examples of small globular proteins (Fig. 1A). ZAG is a 31.5 kDa protein with a class I major histocompatibility complex heavy chain-like fold and a separation distance between the N- and C-termini of ∼45 Å. β2m is a stable 12 kDa protein with an immunoglobulin constant region-like fold that forms a rigid structure with a separation distance between the N- and C-terminus of ∼35 Å (Trinh *et al*., 2002). Likewise, Ub is a compact, stable 8.5 kDa protein with an N- and C-terminal separation distance of ∼37 Å (Vijay-Kumar *et al*., 1987). In addition to the structured linkers chosen from the PDB, proline-rich linkers were designed from the hinge sequence from IgA1 (polyPro and polyPro(Glyc)). This glycosylated region confers rotational flexibility of the Fab relative to the Fc in the context of wild-type dimeric IgA1 (Bonner *et al*., 2008). In addition, glycosylation has been shown to potentially increase stability of polypeptide linkers (Imperiali and O'Connor, 1999). ZAG, β2m and Ub proteins were joined in various combinations with short linker regions, either (Gly_2_Ser)*_n_* repeats, glycosylated proline-rich sequences (polypro(Glyc), or unglycosylated proline-rich sequences (polypro), to create linkers L1–L12 (Table I).

**Table I.** Description of structured linker designs

Linker	Name	Description
L1	GPcPcPc	GlySer-polyPro(Glyc)-polyPro(Glyc)-polyPro(Glyc)
L2	GPPcP	GlySer-polyPro-polyPro(Glyc)-polyPro
L3	GPGcP	GlySer-polyPro-GlySer(Glyc)-polyPro
L4	GPPP	GlySer-polyPro-polyPro-polyPro
L5	GPbP	GlySer-polyPro-β2m-polyPro
L6	GPbG	GlySer-polyPro-β2m-GlySer
L7	PbGbG	polyPro-β2m-GlySer-β2m-GlySer
L8	GPbGbP	GlySer-polyPro-β2m-GlySer-β2m-polyPro
L9	GPUG	GlySer-polyPro-Ub-GlySer
L10	GPZP	GlySer-polyPro-ZAG-polyPro
L11	GGZGZP	GlySer-GlySer-ZAG-GlySer-ZAG-polyPro
L12	GcGcP	GlySer(Glyc)-GlySer(Glyc)-polyPro
L13	cTPR3	(G_4_S)_3_-cTPR3-(G_4_S)_3_
L14	cTPR6	(G_4_S)_3_-cTPR6-(G_4_S)_3_
L15	cTPR9	(G_4_S)_3_-cTPR9-(G_4_S)_3_
L16	cTPR12	(G_4_S)_3_-cTPR12-(G_4_S)_3_
L17	GS1	(G_4_S)_1_
L18	GS2	(G_4_S)_2_
L19	GS3	(G_4_S)_3_
L20	GS5	(G_4_S)_5_
L21	GS6	(G_4_S)_6_
L22	GS7	(G_4_S)_7_
L23	GS8	(G_4_S)_8_
L24	GS9	(G_4_S)_9_

(Gly_4_Ser)*_n_*, Gly–Gly–Gly–Gly–Ser sequence with *n* number of repeats; GlySer, (N-term: AGS(GGS)_3_; Middle: (GGS)_4_; C-term: (GGS)_3_GAS]_2_S); GlySer(Glyc), Gly–Gly–Ser sequence with an embedded potential N-linked glycosylation site (Asn–Ser–Ser); polyPro, proline-rich hinge sequence from IgA1; polyPro(Glyc), proline-rich hinge sequence from IgA1 with an embedded potential N-linked glycosylation site (Asn–Ser–Ser); β2m, β2-microglobulin; Ub, ubiquitin; ZAG, Zn-α2-glycoprotein; cTPRX, consensus tetratricopeptide repeat sequence with X number of repeats.

We also created linkers using tetratricopeptide repeat domains (TPRs; PDB code: 2AVP; L13–L16; Table I; Fig. 1A) (Kajander *et al*., 2007) that are found in natural proteins such as HSP70/90 (Scheufler *et al*., 2000). These domains are optimal for use as potential structured linkers because the length of a set of tandem TPR domains corresponds predictably with the number of repeats. Each repeat consists of 34 amino acids with a defined sequence motif that forms two α-helices (D'Andrea and Regan, 2003). Seven to eight TPRs form a complete superhelical turn with a pitch of ∼72 Å. For our TPR linkers, we used a consensus sequence defined by the amino acid of the greatest global propensity in the natural database of the TPR domains at each position, which was shown to form a stable superhelix and was therefore named the consensus TPR sequence or cTPR (Main *et al*., 2003).

Finally, for comparison, we constructed a series of (Gly_4_Ser)*_n_* linkers (L17–L24; Table I) in order to determine the effect of increasing the number of flexible Gly_4_Ser repeats on the hydrodynamic radius of the IgG. The complete sequence of each linker is given in Table II.

**Table II.** Complete sequences of designed linkers

Linker	Name	Complete sequence
L1	GPcPcPc	AGSGGSGGSGGSPVPSTPPTNSSSTPPTPSPSPVPSTPPTNSSSTPPTPSPSPVPSTPPTNSSSTPPTPSPSAS
L2	GPPcP	AGSGGSGGSGGSPVPSTPPTPSPSTPPTPSPSPVPSTPPTNSSSTPPTPSPSPVPSTPPTPSPSTPPTPSPSAS
L3	GPGcP	AGSGGSGGSGGSPVPSTPPTPSPSTPPTPSPSGGSGNSSGSGGSPVPSTPPTPSPSTPPTPSPSAS
L4	GPPP	AGSGGSGGSGGSPVPSTPPTPSPSTPPTPSPSPVPSTPPTPSPSTPPTPSPSPVPSTPPTPSPSTPPTPSPSAS
L5	GPbP	AGSGGSGGSGGSPVPSTPPTPSPSTPPTPSPSIQRTPKIQVYSRHPAENGKSNFLNCYVSGFHPSDIEVDLLKNGERIEKVEHSDLSFSK DWSFYLLYYTEFTPTEKDEYACRVNHVTLSQPKIVKWDRDPVPSTPPTPSPSTPPTPSPSAS
L6	GPbG	AGSGGSGGSGGSPVPSTPPTPSPSTPPTPSPSIQRTPKIQVYSRHPAENGKSNFLNCYVSGFHPSDIEVDLLKNGERIEKVEHSDLSFS KDWSFYLLYYTEFTPTEKDEYACRVNHVTLSQPKIVKWDRDGGSGGSGGSGGSAS
L7	PbGbG	AGPVPSTPPTPSPSTPPTPSPSIQRTPKIQVYSRHPAENGKSNFLNCYVSGFHPSDIEVDLLKNGERIEKVEHSDLSFSKDWSFYLLYY TEFTPTEKDEYACRVNHVTLSQPKIVKWDRDGGSGGSGGSGGSIQRTPKIQVYSRHPAENGKSNFLNCYVSGFHPSDIEVDLLKNG ERIEKVEHSDLSFSKDWSFYLLYYTEFTPTEKDEYACRVNHVTLSQPKIVKWDRDGGSGGSGGSGAS
L8	GPbGbP	AGSGGSGGSGGSPVPSTPPTPSPSTPPTPSPSIQRTPKIQVYSRHPAENGKSNFLNCYVSGFHPSDIEVDLLKNGERIEKVEHSDLSFSK DWSFYLLYYTEFTPTEKDEYACRVNHVTLSQPKIVKWDRDGGSGGSGGSGGSIQRTPKIQVYSRHPAENGKSNFLNCYVSGFHPSD IEVDLLKNGERIEKVEHSDLSFSKDWSFYLLYYTEFTPTEKDEYACRVNHVTLSQPKIVKWDRDPVPSTPPTPSPSTPPTPSPSAS
L9	GPUG	AGSGGSGGSGGSPVPSTPPTPSPSTPPTPSPSQIFVKTLTGKTITLEVEPSDTIENVKAKIQDKEGIPPDQQRLIFAGKQLEDGRTLSDY NIQKESTLHLVLRLRGGGGSGGSGGSGGSAS
L10	GPZP	AGSGGSGGSGGSPVPSTPPTPSPSTPPTPSPSDGRYSLTYIYTGLSKHVEDVPAFQALGSLNDLQFFRYNSKDRKSQPMGLWRQVE GMEDWKQDSQLQKAREDIFMETLKDIVEYYNDSNGSHVLQGRFGCEIENNRSSGAFWKYYYDGKDYIEFNKEIPAWVPFDPAAQIT KQKWEAEPVYVQRAKAYLEEECPATLRKYLKYSKNILDRQDPPSVVVTSHQAPGEKKKLKCLAYDFYPGKIDVHWTRAGEVQE PELRGDVLHNGNGTYQSWVVVAVPPQDTAPYSCHVQHSSLAQPLVVPWEASPVPSTPPTPSPSTPPTPSAS
L11	GGZGZP	AGSGGSGGSGGSGGSGGSGGSGGSDGRYSLTYIYTGLSKHVEDVPAFQALGSLNDLQFFRYNSKDRKSQPMGLWRQVEGMEDW KQDSQLQKAREDIFMETLKDIVEYYNDSNGSHVLQGRFGCEIENNRSSGAFWKYYYDGKDYIEFNKEIPAWVPFDPAAQITKQKW EAEPVYVQRAKAYLEEECPATLRKYLKYSKNILDRQDPPSVVVTSHQAPGEKKKLKCLAYDFYPGKIDVHWTRAGEVQEPELRGD VLHNGNGTYQSWVVVAVPPQDTAPYSCHVQHSSLAQPLVVPWEASGGSGGSGGSGGSDGRYSLTYIYTGLSKHVEDVPAFQALG SLNDLQFFRYNSKDRKSQPMGLWRQVEGMEDWKQDSQLQKAREDIFMETLKDIVEYYNDSNGSHVLQGRFGCEIENNRSSGAFW KYYYDGKDYIEFNKEIPAWVPFDPAAQITKQKWEAEPVYVQRAKAYLEEECPATLRKYLKYSKNILDRQDPPSVVVTSHQAPGEK KKLKCLAYDFYPGKIDVHWTRAGEVQEPELRGDVLHNGNGTYQSWVVVAVPPQDTAPYSCHVQHSSLAQPLVVPWEASPVPSTP PTPSPSTPPTPSPSAS
L12	GcGcP	AGSGNSSGSGGSGGSGNSSGSGGSPVPSTPPTPSPSTPPTPSPSAS
L13	cTPR3	KLSGGGGSGGGGSGGGGSAEAWYNLGNAYYKQGDYQKAIEYYQKALELDPNNAEAWYNLGNAYYKQGDYQKAIEYYQKALEL DPNNAEAWYNLGNAYYKQGDYQKAIEDYQKALELDPNNLQRSAGGGGSGGGGSGGGGAS
L14	cTPR6	KLSGGGGSGGGGSGGGGSAEAWYNLGNAYYKQGDYQKAIEYYQKALELDPNNAEAWYNLGNAYYKQGDYQKAIEYYQKALE LDPNNAEAWYNLGNAYYKQGDYQKAIEDYQKALELDPNNLQAEAWKNLGNAYYKQGDYQKAIEYYQKALELDPNNASAWYNL GNAYYKQGDYQKAIEYYQKALELDPNNAKAWYRRGNAYYKQGDYQKAIEDYQKALELDPNNRSRSAGGGGSGGGGSGGGGAS
L15	cTPR9	KLSGGGGSGGGGSGGGGSAEAWYNLGNAYYKQGDYQKAIEYYQKALELDPNNAEAWYNLGNAYYKQGDYQKAIEYYQKALEL DPNNAEAWYNLGNAYYKQGDYQKAIEDYQKALELDPNNLQAEAWKNLGNAYYKQGDYQKAIEYYQKALELDPNNASAWYNLG NAYYKQGDYQKAIEYYQKALELDPNNAKAWYRRGNAYYKQGDYQKAIEDYQKALELDPNNRSAEAWYNLGNAYYKQGDYQK AIEYYQKALELDPNNAEAWYNLGNAYYKQGDYQKAIEYYQKALELDPNNAEAWYNLGNAYYKQGDYQKAIEDYQKALELDPN NLQRSAGGGGSGGGGSGGGGAS
L16	cTPR12	KLSGGGGSGGGGSGGGGSAEAWYNLGNAYYKQGDYQKAIEYYQKALELDPNNAEAWYNLGNAYYKQGDYQKAIEYYQKALEL DPNNAEAWYNLGNAYYKQGDYQKAIEDYQKALELDPNNLQAEAWKNLGNAYYKQGDYQKAIEYYQKALELDPNNASAWYNLG NAYYKQGDYQKAIEYYQKALELDPNNAKAWYRRGNAYYKQGDYQKAIEDYQKALELDPNNRSAEAWYNLGNAYYKQGDYQK AIEYYQKALELDPNNAEAWYNLGNAYYKQGDYQKAIEYYQKALELDPNNAEAWYNLGNAYYKQGDYQKAIEDYQKALELDPN NLQAEAWKNLGNAYYKQGDYQKAIEYYQKALELDPNNASAWYNLGNAYYKQGDYQKAIEYYQKALELDPNNAKAWYRRGNAY YKQGDYQKAIEDYQKALELDPNNRSAGGGGSGGGGSGGGGAS
L17	GS1	GGGGSAS
L18	GS2	GGGGSGGGGSAS
L19	GS3	GGGGSGGGGSGGGGSAS
L20	GS5	GGGGSGGGGSGGGGSGGGGSGGGGSAS
L21	GS6	GGGGSGGGGSGGGGSGGGGSGGGGSGGGGSAS
L22	GS7	AGGGSGGGGSGGGGSGGGGSGGGGSGGGGSGGGGSAS
L23	GS8	AGGGSGGGGSGGGGSGGGGSGGGGSGGGGSGGGGSGGGGSAS
L24	GS9	AGGGSGGGGSGGGGSGGGGSGGGGSGGGGSGGGGSGGGGSGGGGSAS

Linkers L1–L21 were inserted into the hinge region of b12 IgG between residues His235 and Thr236. Linkers L22–L24 were inserted into the same hinge between residues Cys231 and Asp232.

As a scaffold for comparing the designed structured linkers, we inserted each into the hinge region of an intact IgG antibody (the anti-HIV antibody b12) (Roben *et al*., 1994). We chose the hinge region of an IgG, which encompasses the amino acids between the C-terminus of the heavy chain portion of the antigen-binding fragment (Fab) and the N-terminus of the Fc, to insert the linkers because it can tolerate large protein insertions (Redpath *et al*., 1998). In addition, extension in the hinge region could potentially increase the separation distance of the Fab arms (Fig. 1B).

### Characterization of the IgGs containing structured linkers

**Fig. 2.**
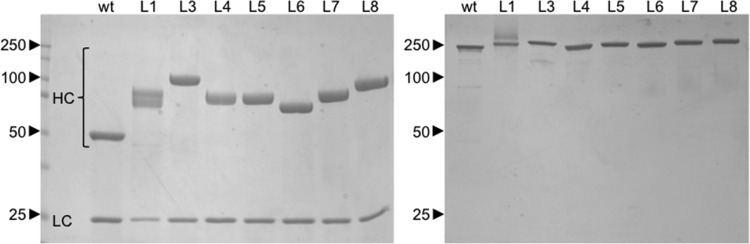
SDS–PAGE analysis of b12 IgG-structured linker proteins run under reducing (left) and non-reducing (right) conditions.

**Fig. 3.**
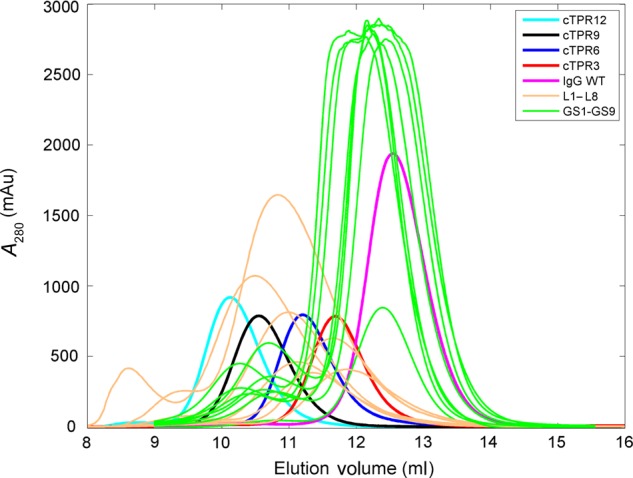
Overlay of size-exclusion chromatograms for IgGs containing flexible and structured protein linkers. Structured linkers (L1–L8) exhibited larger decreases in retention volume with respect to wild-type compared with Gly_4_Ser linkers, which exhibited little to no decrease depending on the number of repeats. Structured cTPR linkers also exhibited consistent decreases in retention volume as a function of the number of repeats.

The b12 IgG proteins containing linkers L1–L24 were expressed by transient transfection in HEK 293-6E mammalian cells and purified by affinity and size exclusion chromatography. Visualization by sodium dodecyl sulfate–polyacrylamide gel electrophoresis (SDS–PAGE) for IgGs containing the L1–L8 linkers showed that all proteins were purified to >95% homogeneity (Fig. 2). Under reducing conditions, two heavy chain bands were observed for b12-L1, which contained a linker containing three potential N-linked glycosylation sites, indicating the presence of multiple glycosylated isoforms. An overlay of the chromatograms derived from SEC showed that the IgGs containing the L1–L8 structured linkers all exhibited a decrease in retention volume relative to wild-type IgG, consistent with the expected increases in the radius of gyration (*R*_g_) of each of the constructs due to the addition of a structured linker (Fig. 3).

We next derived the hydrodynamic radii using DLS for wild-type b12 and the b12 proteins containing designed linkers. DLS measures fluctuations in the intensity of scattered light of a protein solution over time, which can be used to calculate an autocorrelation function of intensity (Nobbmann *et al*., 2007). Typical monodisperse samples (including our hinge-linked antibodies) generate an exponential decay in the autocorrelation. A least squares fit can be performed to calculate the decay constant, which directly relates to the diffusion coefficient. The diffusion coefficient is then inversely related to the characteristic hydrodynamic radius *R*_H_, which reflects the radius of a hypothetical solid sphere that would diffuse at the same rate as the protein. The *R*_H_ value is not a direct measurement of the length that the linker contributes to the size of the IgG. However, comparative analysis can yield rank order differences for the relative lengths and rigidity of the various linkers. For example, if the separation between the IgG Fc and Fab domains were increased by the addition of a designed hinge linker, we would expect an observable increase in the *R*_H_ of the fusion construct compared with the parental b12 IgG due to increased size of the diffusion sphere.

**Fig. 4.**
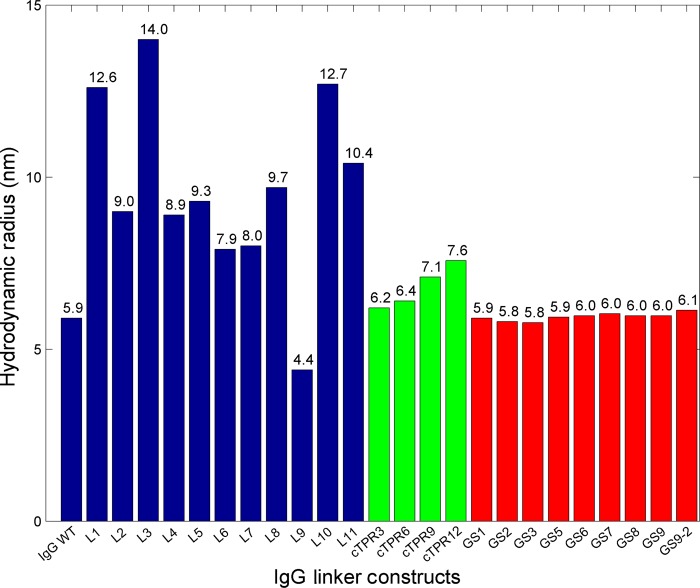
Comparative analysis by DLS of the hydrodynamic radii (*R*_H_) of designed linkers in the context of the b12 IgG.

The hydrodynamic radii were measured by DLS for each of the b12 IgG-linker fusion proteins and compared with an internal wild-type b12 IgG control (Fig. 4). By comparing constructs containing elongated or small protein domain linkers, cTPR repeat linkers and flexible (Gly_4_Ser)*_n_* linkers of various lengths (L17–L24), we could directly compare the effects of incorporating different lengths of flexible vs. structured proteins linkers.

We observed a consistent trend for the *R*_H_ values between glycosylated and non-glycosylated linkers (L1, L2, L3 and L12 vs. L4). The incorporation of three potential N-linked glycosylation sites in proline-rich linkers derived from the hinge region of IgA1 (L1) appeared to increase the *R*_H_ relative to constructs containing similar linker sequences with only one (L2) or no (L4) N-linked glycosylation sites, possibly through stabilization of the folded state and leading the linker to adopt a more extended conformation (Shental-Bechor and Levy, 2008). While the addition of only a single potential N-linked glycosylation site did not seem to affect the diffusion rate of proline-rich linkers (compare L2 and L4), a single potential N-linked glycosylation in the GGSG-NSS-GSGG region of a combination proline-rich and Gly_2_Ser linker (L3) increased its *R*_H_ beyond the *R*_H_ of a proline-rich linker with three potential N-linked glycosylation sites (L1). These data are consistent with the observation that N-linked glycosylation confers rigidity in the backbone of a flexible linker (Liu *et al*., 2000), suggesting these reagents contained linkers with a more extended conformation. Thus incorporating potential N-linked glycosylation sites within flexible linkers may be a general method to increase linker rigidity.

Adding a single β2m domain to a linker increased the *R*_H_ of the b12-linker protein to a similar degree as a proline-rich repeat relative to IgG (compare L5 to L2, L4 and IgG), suggesting that the structured β2m domain provided similar bulk and separation to the polyPro repeat. However, adding a second tandem β2m repeat separated from the first with a (Gly_2_Ser)_4_ sequence did not increase the *R*_H_ appreciably (compare L8 and L5; L7 and L6). These results suggested that coupling a flexible Gly–Ser linker with the rigid β2m domain partially diminished the separation between Fc and Fab regions provided by β2m alone. A similar observation was made for hinge constructs containing ZAG (L10 and L11). A linker containing ZAG alone increased the hydrodynamic radius of the b12-linker protein compared with IgG and more than the IgG–proline-rich linker (compare L10 to L2 and L4). However, replacement of the proline-rich domain by ZAG that was flanked at both termini by a (G_2_S)_4_ peptide resulted in a decrease in hydrodynamic radius (compare L10 and L11).

We also investigated ubiquitin as a structured linker (L9). However, initial characterization by SDS–PAGE showed degradation at the linker site (data not shown). In addition, DLS measurements revealed that a purified sample of b12-L9 had a smaller *R*_H_ than IgG similar to a Fab or Fc region alone, further suggesting ubiquitin-specific degradation (Fig. 4).

### cTPR linker series

cTPR constructs were generated with 3, 6, 9 or 12 tandem repeats. All cTPR linkers were flanked by (Gly_4_Ser)_3_ sequences (Table II). The constructs exhibited a consistent decrease in elution volume on SEC as a function of the repeat length (Fig. 3). These constructs also predictably increased the *R*_H_ of the linked IgG with increased number of tandem repeats (Fig. 4). The hydrodynamic radius of the cTPR12 construct corresponded to approximately the size of L4, which contained a proline-rich linker. These data suggested that, unlike with repeated domains of the structured linkers, the increase in separation between the Fab and Fc correlated predictably with the number of cTPR repeats despite the presence of Gly_4_Ser peptides flanking the N- and C-termini.

### (Gly_4_ser)*_n_* linker series

In order to compare our structured linkers to the typical unstructured Gly–Ser linkers commonly used in protein design and engineering, we constructed, expressed and purified eight IgG-(G_4_S)*_n_* variants. In contrast to the SEC profiles for the structured linker constructs, there were only small differences in elution volume for the IgGs including Gly_4_Ser linkers (L17–L24). These differences often did not correlate with molecular mass as IgG-GS9, the IgG with the largest linker, eluted at approximately the same volume as wild-type IgG, which eluted after some of the constructs with shorter linkers (Fig. 3).

Unlike proline-rich linkers and rigid linkers consisting of natural protein domains such as β2m, Gly_4_Ser linkers that did not contain a potential N-linked glycosylation site did not detectably increase the hydrodynamic radius of the IgG, suggesting that these linkers did not provide increased separation between the Fab and Fc domains (Fig. 4). These results were consistent with the observation that Gly_4_Ser linkers did not provide significant separation between the joined domains in the context of other fusion proteins (Arai *et al.*, 2001). Measurements of IgG-GS9 from two preparations showed only a slight difference in *R*_H_ (0.1 nm), indicating that these measurements were quite robust and relatively small differences in *R*_H_ may be significant.

Optimized linkers are important for the construction of multi-functional fusion proteins, in terms of both immunogenicity and conformational dynamics. Different linker compositions can alter their effective length and rigidity. In this study, we used SEC and DLS to characterize designed linkers in the context of an IgG to determine whether these linkers could increase the distance between the antigen-binding fragments. We found that flexible Gly_4_Ser linkers did not increase the *R*_H_ of fused reagents, suggesting these linkers did not provide increased separation between the Fab and Fc domains even with up to nine Gly_4_Ser repeats, in agreement with previous studies (Arai *et al*., 2001). By contrast, the structured helical cTPR linkers provided consistent increases in *R*_H_ and SEC elution volume as a function of repeat number, indicating that these repeats can be used to increase the separation distance between two proteins or domains. Our other designed linkers, including those containing naturally occurring proteins such as β2m and ZAG, yielded increases in the observed *R*_H_ by as much as twice the *R*_H_ of a naturally occurring IgG. The systematic characterization of the lengths and rigidity properties of the structured protein linkers and a range of (Gly_4_Ser)*_n_* linkers reported here provide a new set of tools to the available linker repertoire for engineering fusion proteins.

## Funding

This work was supported by a grant from the Bill and Melinda Gates Foundation through the Grand Challenges in Global Health Initiative, the Director's Pioneer Award [1DP1OD006961-01 to P.J.B.] and the National Institutes of Health HIVRAD [P01Al100148 to P.J.B.]. Funding to pay the Open Access publication charges for this article was provided by NIDA/National Institutes of Health.
